# Rupture access to hydrous minerals controls aftershocks in subduction zones

**DOI:** 10.1038/s41598-026-38159-6

**Published:** 2026-02-10

**Authors:** Thanushika Gunatilake, Taras Gerya, James A. D. Connolly, Stephen A. Miller

**Affiliations:** 1 University of Neuchâtel, The Centre for Hydrogeology and Geothermics (CHYN), Rue Emille Argand 8, 2000 Neuchâtel, Switzerland; 2 ETH Zürich, Department of Earth Sciences, Sonnengesstrasse 5, 8006 Zürich, Switzerland

**Keywords:** Aftershocks, No aftershocks, Fluids in subduction, Thermal decomposition, Dehydration, Hydrated minerals, Natural hazards, Solid Earth sciences

## Abstract

Aftershock productivity varies widely among subduction-zone earthquakes of similar magnitude. We investigate how slab hydration and rupture geometry modulate access to hydrous minerals and the generation of pressurized fluids from co-seismic frictional heating along the interface between subducting slabs and overriding plates. We describe ten large and major earthquakes (Mw > 6.8) that generated thousands of aftershocks (Mw > 4), and eleven nearby earthquakes of similar magnitude that generated few, if any, aftershocks. Kinematic and petrological constraints reveal that earthquakes producing rich aftershock sequences ruptured along slab interfaces containing serpentinized peridotite and hydrated oceanic crust. By contrast, earthquakes with few aftershocks occurred in flat-slab regions where rupture planes were oblique to the hydrated interface (intraslab events). Oblique rupture reduces the volume of volatile-bearing minerals accessed per unit rupture area, diminishing the pressurized fluid production needed to drive aftershock sequences. Globally, we propose that slab geometry and rupture orientation regulate access to fluid-producing hydrated rocks and thereby control aftershock productivity through co-seismically generated pressurized fluids in subduction zones.

## Introduction

The physical driver responsible for aftershocks has remained elusive since 1895 when Omori recognized that the rate of aftershocks decays as 1/time^1^. Several empirical relationships describe earthquake statistical properties, including the Omori-Utsu, Gutenberg-Richter, and Bath Laws^2–6^. The Omori-Utsu law describes the temporal decay of aftershocks following a mainshock, the Gutenberg Richter law characterizes the frequency-magnitude distribution of earthquakes, and Bath’s Law states that the maximum aftershock is at least one magnitude smaller than the mainshock. A widely-used statistical model, the Epidemic Type Aftershock Sequence (ETAS), is a stochastic point process model that allows events to generate daughter events, but to date lacks a physical basis. Aftershock productivity, one of the terms in the Omori-Utsu Law, varies widely among earthquakes of the same magnitude, sometimes by orders of magnitude. Observationally, aftershock productivity, the number of aftershocks triggered by a mainshock, for intermediate (70-300km) and deep earthquakes (>300km) is low compared to shallow earthquakes (<70km)^7^, with some evidence that seismicity rates, reflecting the long-term frequency of earthquake occurrence, are coupled to the degree of hydration of the incoming plate^8^.

Hydration of the downgoing plate occurs through multiple processes, including infiltration of seawater along outer-rise faults prior to subduction and progressive metamorphic reactions within the oceanic crust and upper mantle during subduction^9,10^. As a result, both the slab interface and the slab interior may contain significant volumes of hydrous minerals. However, global geodynamic and petrological models show that steep subduction preserves a continuous, mineralogically hydrated shear zone along the interface, whereas flat-slab segments tend to be thermally warmer and exhibit reduced and discontinuous hydration^10,11^. These geometrical and thermal differences strongly influence the availability and spatial distribution of fluid-producing lithologies. Miller 2020^12^ proposed that fluid pressure diffusion controlled by permeability dynamics defines the decay rate of aftershocks, with permeability controlled by the orientation of the causative fault relative to the prevailing regional stress field. Aftershocks propagate faster in regions with higher permeability^13^, such as fractures, where fluid movement reduces the effective normal stress on the fault and thereby facilitates faster slip^14^, while in less permeable areas, fluid movement is restricted, leading to slower propagation^15^. These factors, in combination, contribute to spatial variations in fluid distribution along the subduction interface. Areas with more favorable conditions for fluid retention may store larger amounts of pre-existing fluids^16^, while other regions may have limited fluid accumulation. As a result, the distribution of pre-existing fluids within the system is highly heterogeneous. Recent investigations by Gunatilake and Miller (2022, 2024) and Gunatilake (2023) showed that thermal decomposition of minerals plays a dominant role in driving aftershock sequences in the carbonate system of the Apennines^17–19^. In addition, other studies established that large earthquakes occurring during subduction are associated with fluid pressure variations^20^ and further demonstrated the role of serpentine dehydration in triggering episodic tremor and slip (ETS) events at intermediate depths within subduction zones^21–23^. Interestingly, Kawakatsu and Seno^24^ documented regional variations in seismicity along the northern Honshu arc that correlate with changes in subduction angle. Their work established an important observational link between slab geometry and seismic behavior.

In this study, we extend this observation by exploring a physical mechanism—rupture access to hydrated lithologies, that may explain the contrasting aftershock productivity across different subduction geometries. Several global studies have demonstrated that slab dip (steep vs. flat subduction) exerts a first-order control on subduction zone seismic behavior^25,26^. Here we extend this geometrical perspective by examining how slab dip regulates access to hydrated lithologies and, consequently, aftershock productivity. We define steep subduction as typical subduction zones with a monotonically increasing depth-to-slab, while flat subduction shows very shallow dip of oceanic crust within the subduction zone. Flat subduction occurs along 10$\%$ of modern convergent margins^27,28^, and is associated with elevated mantle temperatures, younger crust, thicker oceanic crust, and depleted mantle^29,30^. Steep subduction zones commonly preserve a continuous, mechanically weak, and strongly hydrated plate interface containing serpentinized mantle and hydrothermally altered oceanic crust^10,11^. During rapid coseismic slip, shear heating along this hydrated interface can trigger thermal dehydration, producing substantial volumes of high-pressure fluids capable of sustaining prolonged aftershock sequences^31–35^. In contrast, flat-slab geometry disrupts or truncates the hydrated plate-interface shear zone, and the overall hydration state of the slab is significantly reduced compared with steep subduction^34,36^. As a result, earthquakes in flat-slab settings more often rupture along faults that intersect the interface at high angles and encounter only small, discontinuous volumes of hydrous minerals. These ruptures access far less hydrous material than interface-parallel events in steep slabs, thereby limiting coseismic devolatilization and greatly reducing aftershock productivity. We recognize that stress perturbations from slip on the fault can contribute to early aftershocks^37^ and that viscoelastic relaxation, aseismic slip, and velocity-strengthening regions may also influence short-term postseismic behavior^38^. However, these mechanisms generally decay rapidly in time, whereas our analysis focuses on long-lived aftershock sequences that require a sustained source of overpressured fluids.

**Fig. 1:**
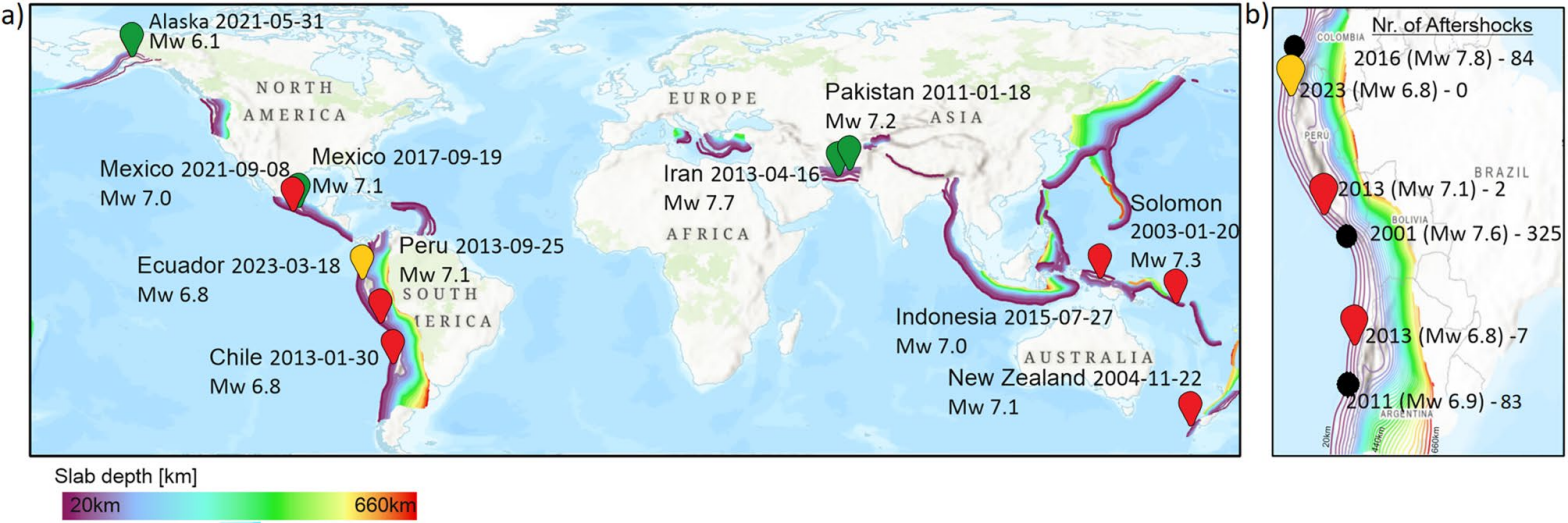
Conceptual framework. (**a**) Location of the eleven large and major earthquakes (Mw>6.8). The green (normal), red (thrust), and yellow (strike-slip) symbols indicate earthquakes that generated generated few, if any, aftershocks with Slab depths superposed^39^. (**b**) Expanded view of the South American subduction system showing two regions of flat and steep subduction in Peru and Chile. Black circles represent large and major earthquakes that generated numerous aftershocks for similar magnitudes. Maps were generated by the authors using ArcGIS Pro (v3.1; https://www.esri.com/arcgis-pro).

Although fluids are widely recognized as an important factor influencing aftershock productivity^40–44^, it remains unclear why earthquakes of similar magnitude may access very different fluid budgets. The processes controlling fluid availability during rupture, including slab hydration state, rupture geometry, and thermal–mechanical conditions are still debated. In this study, we explore how slab dip and rupture geometry may regulate access to fluid-producing hydrous minerals and thereby influence aftershock productivity. We expand on the observed variability in aftershock productivity by examining contrasting tectonic and petrological environments. We analyzed recent large and major subduction-related earthquakes to investigate how slab dip and rupture geometry influence aftershock productivity. We compare aftershock behavior in both flat and steep subduction settings using aftershock counts, focal mechanisms of the main shock, and slab interface geometry as constrained by the Slab2.0 model^39^. We limit our study to earthquakes shallower than 70 km to minimize the influence of mechanisms that dominate intermediate- and deep-focus seismicity, which are known to generate fewer aftershocks^7,8^. We acknowledge that different physical processes may control aftershock behavior at greater depths. Within this framework, our results highlight significant differences between earthquakes that rupture along the hydrated plate interface and those that rupture within the slab on planes that intersect the interface at a high angle. In these intraslab cases, the rupture cuts across and away from the hydrated shear zone, propagating into the underlying oceanic crust or mantle and thereby accessing substantially smaller volumes of hydrous minerals.

Figure 1a shows the earthquakes investigated in this study with depth-to-slab contours shown superposed, derived from the Slab2.0 model^39^, whose regional uncertainties are small enough that the first-order geometry and dip are reliably captured. Thrust events are shown in red, normal-faulting events in green, and strike-slip events in yellow. All of these events generated far fewer aftershocks (Mw > 4) than expected. The close-up of the Nazca plate-South American subduction zone (Fig. 1b) shows three significant aftershock-rich sequences (black) in regions with typical (steep) subduction, and three significant aftershock-free earthquakes associated with flat subduction. These examples serve as the basis for this study because we find this pattern on a global scale. We focused on pairs of spatially proximal earthquakes (within the same subduction margin segment) with comparable magnitudes (Mw > 6.5) that occurred on opposite sides of a well-defined steep–to–flat slab transition. This design allows us to isolate the effect of slab geometry on aftershock productivity while minimizing differences in tectonic setting, convergence rate, and lithospheric age. Regions without such clear geometric transitions (e.g., most of the Japan trench) were not included because suitable steep–flat pairs could not be identified there. Following this approach, we investigated 11 large-to-major earthquakes (Mw > 6.8 to Mw = 8) that produced few or no aftershocks, including both interface and intraslab events with thrust, normal, and strike-slip mechanisms, and compared them with ten nearby earthquakes of similar magnitude that generated abundant aftershocks. The global distribution of all earthquakes analyzed in this study is shown in Supplementary Figure S1. We present aftershock density as heat maps of in the three months following the mainshock, with the first three weeks of individual events shown superposed. Heatmaps of the first three months represent the broader spatial distribution of seismicity, while the individual events visualize the immediate response to the mainshock and minimize external influences. The spatial extent of each heat map was chosen based on the observed distribution of seismicity following the mainshock; consequently, the mapped area varies between events and may include distant seismicity that is not interpreted as directly triggered aftershocks. Data was obtained from the International Seismological Centre (ISC), and the US Geological Survey (USGS). The numbers of events (Mw>4) listed were compiled for the first three weeks provided by US Geological Survey (USGS).

## Observations and results

### Chile, Peru, Ecuador

**Fig. 2:**
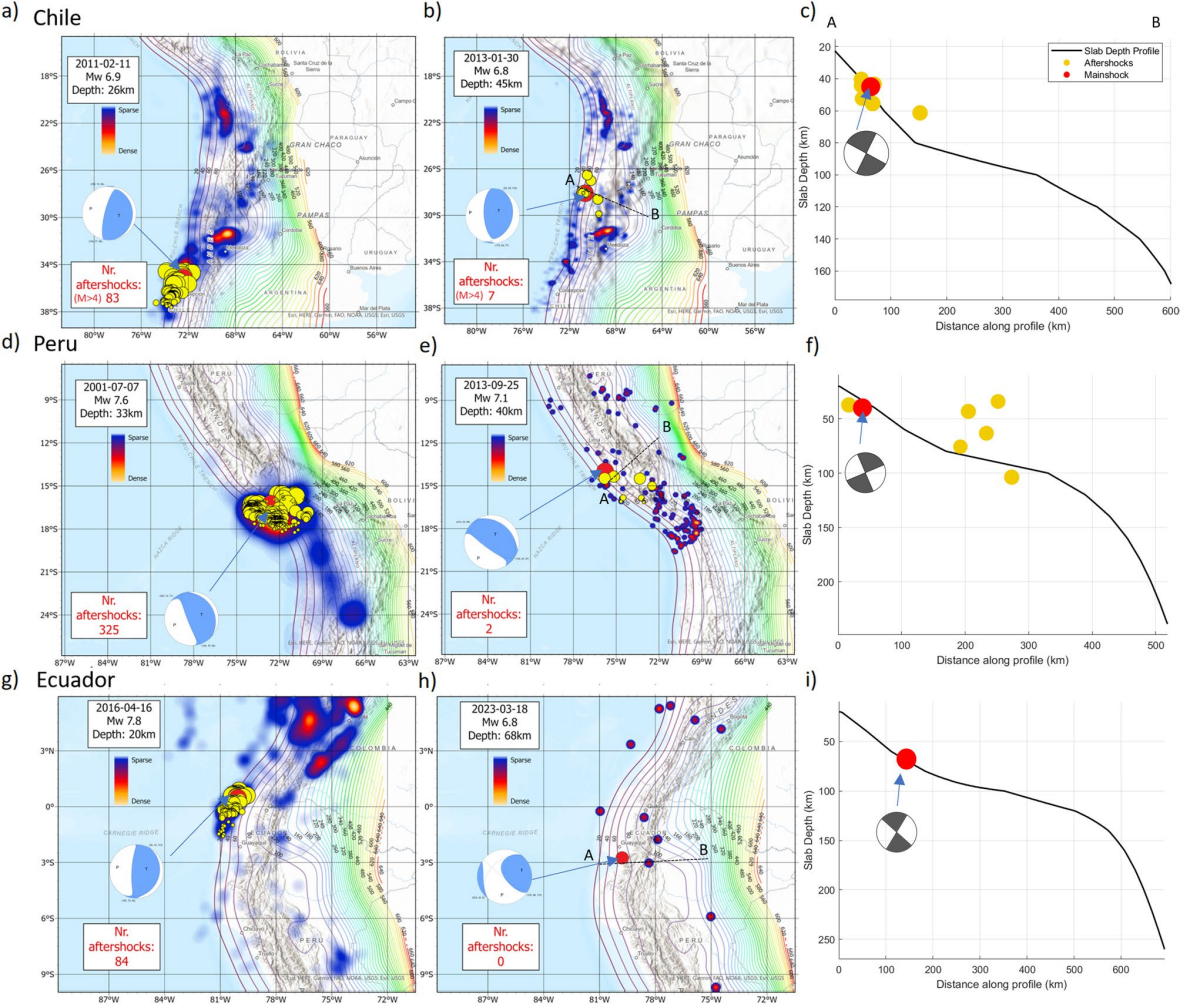
Earthquakes studied: Chile, Peru and Ecuador with slab depth superposed. The red circles represent the mainshock, while the yellow circles denote aftershock events occurring in the first three weeks, specified in red in the box. Panel 1 (**a,d,g**) shows three different large-to-major events generating rich aftershock sequences visualized as heat maps quantifying aftershock density for the first three months. Panel 2 (**b,e,h**), on the other hand, show three large-to-major earthquakes that produced few if any aftershocks. The heat map is generated for 3 months after the mainshock and clearly shows a dearth of events in each case, with no aftershocks (Mw>4) registered for Ecuador. Panel 3 (**c,f,i**) show the slab interface in cross-section (indicated by A-B in Panel 2) of the subduction system. Focal spheres for Chile and Peru (**e,f**) show that both of the fault planes are oblique to the interface. Maps were generated by the authors using ArcGIS Pro (v3.1; https://www.esri.com/arcgis-pro) based on publicly available seismic data from the International Seismological Centre (ISC; https://www.isc.ac.uk/) and slab depth models from the U.S. Geological Survey (Hayes et al., 2018; https://doi.org/10.5066/F7PV6JNV).

Figure 2a shows a typical aftershock sequence of a Mw6.9 subduction zone earthquake in southern Chile (02/2011). The shallow dip (15 degrees) of this thrust fault is consistent with the dip of the subduction zone, indicating rupture of the subduction zone interface. The heat map for this event shows intense aftershock activity with more than one-thousand aftershocks (Mw>4) in the first three months, with 83 aftershocks (Mw>4) in the first three weeks. The cumulative aftershock sequences for these events further highlight this contrast, as shown in Supplementary Figure S4. Figure  2b shows the heat map aftershock density of a Mw6.8 thrust earthquake (01/2013), also in Chile, where only seven aftershocks (Mw>4) were registered in the first 3 weeks. Figure 2c shows the focal sphere at depth of this event superposed on the well-constrained depth-to-slab interface. Note, that focal mechanisms contain a nodal-plane ambiguity, we evaluated both planes for each earthquake relative to the slab-interface geometry. In these aftershock-poor cases, the preferred plane was identified using a combination of aftershock depth patterns, regional slab geometry, and published finite-fault or slip models; where these constraints were insufficient (e.g., complete absence of aftershocks), we report the ambiguity explicitly and present both geometric possibilities. The hypocenter for this event occurred at a depth of 45 km and at the slab interface. The focal sphere shows obliquity to the hydrous interface for both the true fault plane and auxiliary plane. Typically, aftershocks can be used to help constrain the true fault plane, but their limited number and poor location complicates the matter. Although absolute hypocentral depths carry non-negligible uncertainties, the catalogued aftershocks form a coherent cluster that lies systematically deeper than the mainshock. This systematic contrast points to fundamental differences in the stress regime between flat and steep subduction zones. Mechanically, the dominant force driving steep subduction is slab pull from the negative buoyancy of the descending plate. In flat-slab regions, however, the shallow geometry reduces the vertical component of slab pull and redistributes the force horizontally over several hundred kilometers. This configuration increases the transmission of ridge-push forces into the downgoing plate^45,46^, resulting in enhanced horizontal compression within the slab. Such a stress regime can promote thrust faulting on faults that are oriented obliquely to the hydrated interface, consistent with the mechanisms inferred for the flat-slab earthquakes examined here^47^.

Further north (in Peru), Fig. 2d shows intense aftershock activity in the heat map following an Mw7.6 event in July 2001 with 325 aftershocks (Mw>4) in the first three weeks. By contrast, Fig. 2e shows the heat map for a nearby Mw 7.1 earthquake (09/2013), which generated only six aftershocks, three in the first week, and three in the third week. Location accuracy of aftershock hypocenters is uncertain, but the two possible fault planes indicated by the focal mechanism (Fig. 2f) are both oblique to the hydrated interface.

Continuing further north to Ecuador (Fig. 2g), the heat map of aftershock density following the Mw 7.8 megathrust interface earthquake shows 84 aftershocks (Mw > 4) within the first three weeks. In contrast, just south of this event, the Mw 6.8 strike-slip earthquake of March 2023 (Fig. 2h) produced no aftershocks, which is highly anomalous for an event of this magnitude. This earthquake occurred precisely at the transition between steep and flat subduction, a region characterized by strong along-strike gradients in slab geometry. The hypocenter locates directly on the plate interface (Fig. 2i), and the right-lateral focal mechanism exhibits an $\sim$86$^\circ$ dip, indicating a steep, nearly vertical rupture plane that cuts across the interface rather than propagating along it. Such interface-crossing strike-slip faulting is consistent with the mechanical need to accommodate lateral variations in slab kinematics between adjacent segments with different slab dip and convergence behavior^48,49^. The absence of aftershocks despite a moment magnitude of  6.8 is notable and is consistent with rupture geometry that limits access to hydrated material, as predicted for transitions to flat subduction^49,50^.

### Mexico, Iran, Pakistan, Indonesia

**Fig. 3:**
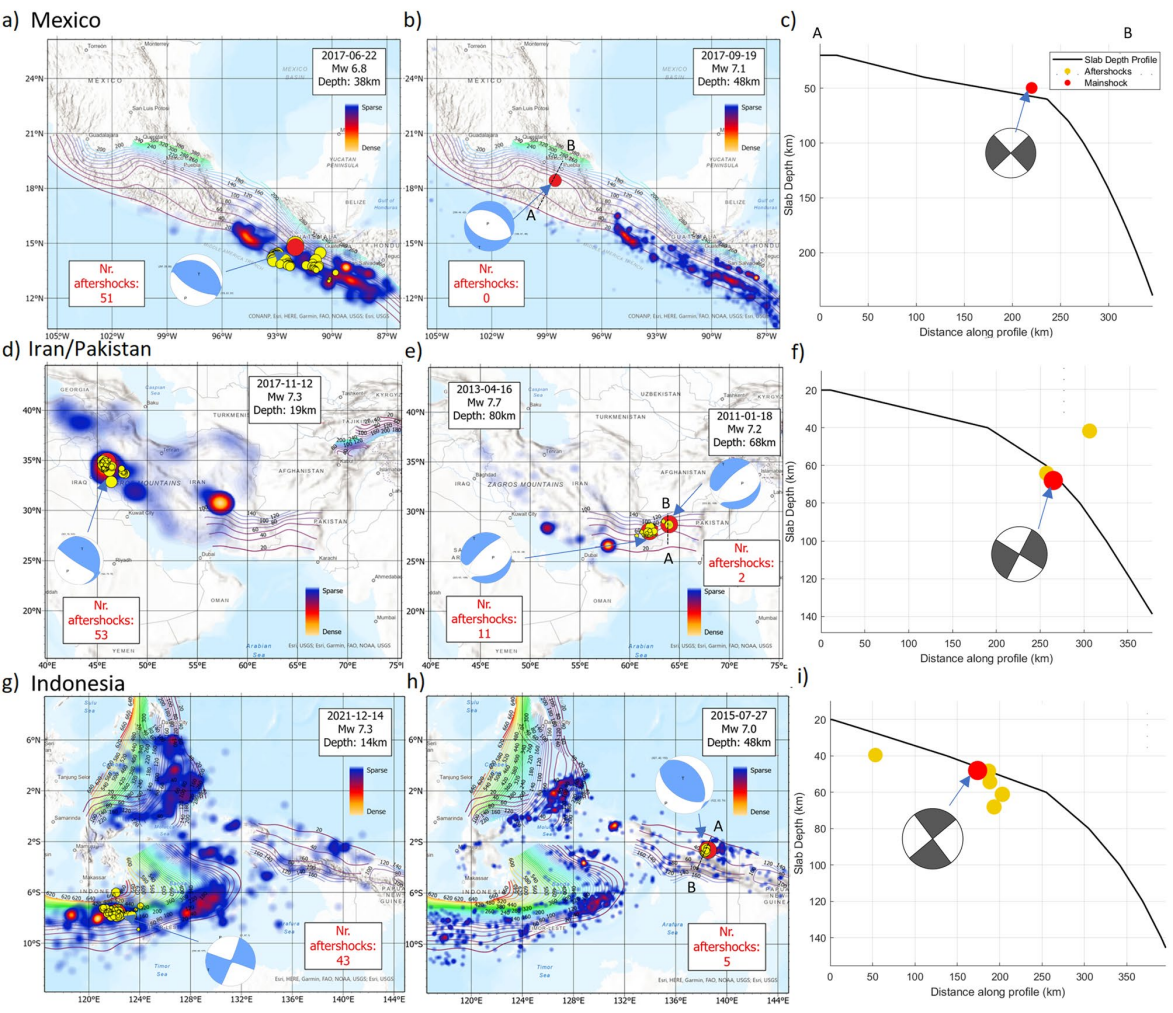
Earthquakes studied: Mexico, Iran, Pakistan and Indonesia with slab depth superposed. The red circles represent the mainshock, while the yellow circles denote aftershock events. Panel 1 (**a,d,g**) shows three different significant events generating rich aftershock sequences visualized as heat maps quantifying aftershock density for the first three months. Panel 2 (**b,e,h**) visualizes three major seismic events that produced few, if any, aftershocks. The heat map is generated for 3 months after the mainshock and clearly shows a dearth of events in each case. Panel 3 (**c,f,i**) shows the profile of the slab interface of the subduction system and the corresponding focal sphere at depth for each large-to-major earthquake event. See text for detailed description.

A consistent pattern begins to emerge. Two earthquakes in Central America exhibited dramatically different behaviors, with an Mw6.8 event spawning 51 (Mw>4.0) aftershocks in the first three weeks (Fig. 3a), while an even larger event (Mw7.1) failed to produce any aftershocks (Fig. 3b). The focal mechanism for aftershock-rich earthquake shows a low-angle thrust fault event, typical for interface-hosting subduction zone earthquakes. The focal mechanism for the larger Mexico event showed pure normal faulting consistent flexural stresses at the transition from flat to very steep subduction. This indicates a return of slab-pull forces and flexure of the subducting slab. Interestingly, a slip model of this earthquake identified the nucleation point of the earthquake to be either at the bottom of the oceanic crust or within the oceanic mantle^51^. Without aftershocks, determining the causative fault is impossible, but both the true and axiliary fault planes ruptured oblique to the hydrated interface. An similar situation was identified^12^ on May 26, 2019 in Peru where a great earthquake (Mw = 8) with a normal-faulting focal mechanism occurred at the transition from flat to steep subduction, and which likewise generated no aftershocks. Although intermediate-depth intraslab normal-faulting earthquakes are also known to produce few aftershocks due to slab-bending stresses and reduced fluid budgets^52^, the Peru event occurred at shallow depth, so its aftershock-poor behavior is more consistent with limited access to hydrated material at the flat–steep slab transition.

In the Zagros Mountains (Iraq) a Mw7.3 thrust earthquake occurred in the Bisotoun carbonate platform, the thickest lithological unit in the region^53^, and generated 53 (Mw>4.0) aftershocks during the first three weeks following the mainshock (Figure 3b, Panel 1). Not coincidentally, the thickest lithological unit in the region is the Bisotoun carbonate platform^53^. In this case, we speculate that decarbonization drove these aftershocks in the same manner that decarbonization drives aftershocks in the Apennines^17–19^. By contrast, farther to the southeast in Iran/Pakistan, two major earthquakes (Mw 7.7 and Mw 7.2) with normal faulting focal mechanisms (Fig. 3e) occurred within the Makran subduction system, which is widely recognised as a low-angle to flat-slab segment^54^. Although the dip is not as shallow as the Chilean flat slab, this region exhibits the characteristic geometric and mechanical features of flat subduction. The larger event generated eleven aftershocks (Mw > 4.0), and the Mw 7.2 event produced only two. Normal-faulting ruptures generally involve lower shear stress and reduced frictional heating than thrust events, limiting coseismic fluid overpressurization and providing an additional explanation for their characteristically low aftershock productivity. The cross-section (Fig. 3f), together with the steep west-dipping nodal plane of the focal mechanism, indicates a rupture that propagated upward and obliquely across the hydrated plate interface.

Indonesia is located in an extremely complex geodynamic setting and displays disparate aftershock behavior. For example, a Mw 7.3 strike-slip earthquake (Fig. 3g) generated 43 aftershocks (Mw > 4.0) in the first three weeks, while farther east, in the Timor Sea, a Mw 7.0 thrust earthquake generated only five aftershocks over the same interval. The Mw 7.0 event is best interpreted as an intraslab earthquake: its hypocenter lies at the depth of the downgoing slab (Fig. 3h), and both possible fault-plane solutions dip obliquely to the hydrated interface^55^ . The aftershocks cluster along the east-dipping nodal plane, indicating rupture propagation into the oceanic lithosphere. This intraslab event reflect reactivation of inherited seafloor structures or hydrated faults^56–58^. This rupture’s obliquity to the hydrated interface implies propagation into the comparatively dry oceanic lithosphere, providing a natural explanation for the very low aftershock productivity observed.

Three additional cases (Alaska, the Solomon Islands, and New Zealand) with results similar to those presented here are found Supplementary Materials (Fig. S2). Although this study focuses on subduction-zone seismicity, we also identify several examples outside subduction settings in which co-seismic devolatilization—-whether through dehydration of hydrous minerals or thermal decomposition of carbonates—drives aftershock sequences that persist well beyond the initial weeks of deformation. For instance, two large earthquakes in 2023 provide additional evidence for co-seismically induced fluid production. For instance, a Mw 6.8 thrust earthquake (Sept. 13, 2023) in Morocco ruptured along an E-W trending fault, generating just five aftershocks in the first week (Mw>4), with no more to follow. In contrast, a much smaller Mw 6.3 thrust fault (October 7, 2023) in Afghanistan ruptured along an E–W trending fault and generated 48 aftershocks (Mw > 4), including five additional Mw 6.3 events. This is almost an order of magnitude more aftershocks for an earthquake half the size. We propose that this stark difference in aftershock behavior is linked to the geological setting. The Afghanistan earthquakes occurred within an extensive carbonate platform, where co-seismic thermal decomposition of carbonates likely contributed to sustained fluid production and prolonged aftershock activity^12,18,59^. In contrast, the Moroccan earthquake ruptured within Variscan and older basement rocks, which contain minimal carbonate or hydrous-bearing mineral deposits, thereby limiting fluid-driven aftershock generation. These examples illustrate that prolonged aftershock sequences can arise from devolatilization in both hydrated interface zones and volumetrically distributed lithologies, and are not restricted to a single structural setting. Across all paired examples, presented in Figs. 2 and 3, the aftershock-poor earthquakes systematically nucleate at greater depths than the corresponding aftershock-productive events. These greater depths occur within flat-slab settings, where the hydrated plate-interface shear zone is reduced or disrupted, suggesting that depth, slab geometry, and rupture orientation covary and jointly influence access to hydrated material.

## Discussion

Hydration and dehydration in subduction zones are volumetric processes that affect both the oceanic crust and the underlying mantle. Prior to subduction, outer-rise faulting allows seawater to penetrate deeply into the oceanic plate, producing hydrated structures that may later be reactivated as intraslab faults^9,60^. Once subduction begins, deformation in steeply dipping systems preferentially localizes along a continuous, mechanically weak, and mineralogically hydrated shear zone at the plate interface. In the shallow forearc (updip of the mantle-wedge corner), this hydrated interface is primarily composed of subducted sediments and altered oceanic crust, whereas farther downdip, serpentinized mantle within the subducting plate becomes the dominant hydrous component. Together, these lithologies form the largest accessible reservoir of hydrous minerals available to shallow and intermediate-depth ruptures. By contrast, ruptures that cut obliquely across the interface, typical of many intraslab earthquakes, intersect far smaller and less continuous hydrated domains, substantially limiting their capacity for coseismic devolatilization and the generation of robust aftershock sequences.

A second key distinction relevant to aftershock variability lies in the contrasting durations of stress-triggered and fluid-driven aftershock sequences. Static and dynamic Coulomb stress changes influence only the earliest stage of aftershock activity, typically hours to a few days, because they result from a single, instantaneous coseismic stress step that does not persist^37,61^. In contrast, sequences that continue for weeks to months are more consistent with pore-pressure diffusion and evolving permeability within hydrated lithologies^62,63^. This fundamental difference in duration provides a clear physical basis for distinguishing short-lived, mechanically triggered sequences from sustained aftershock activity driven by fluid overpressure and dehydration processes.

**Fig. 4:**
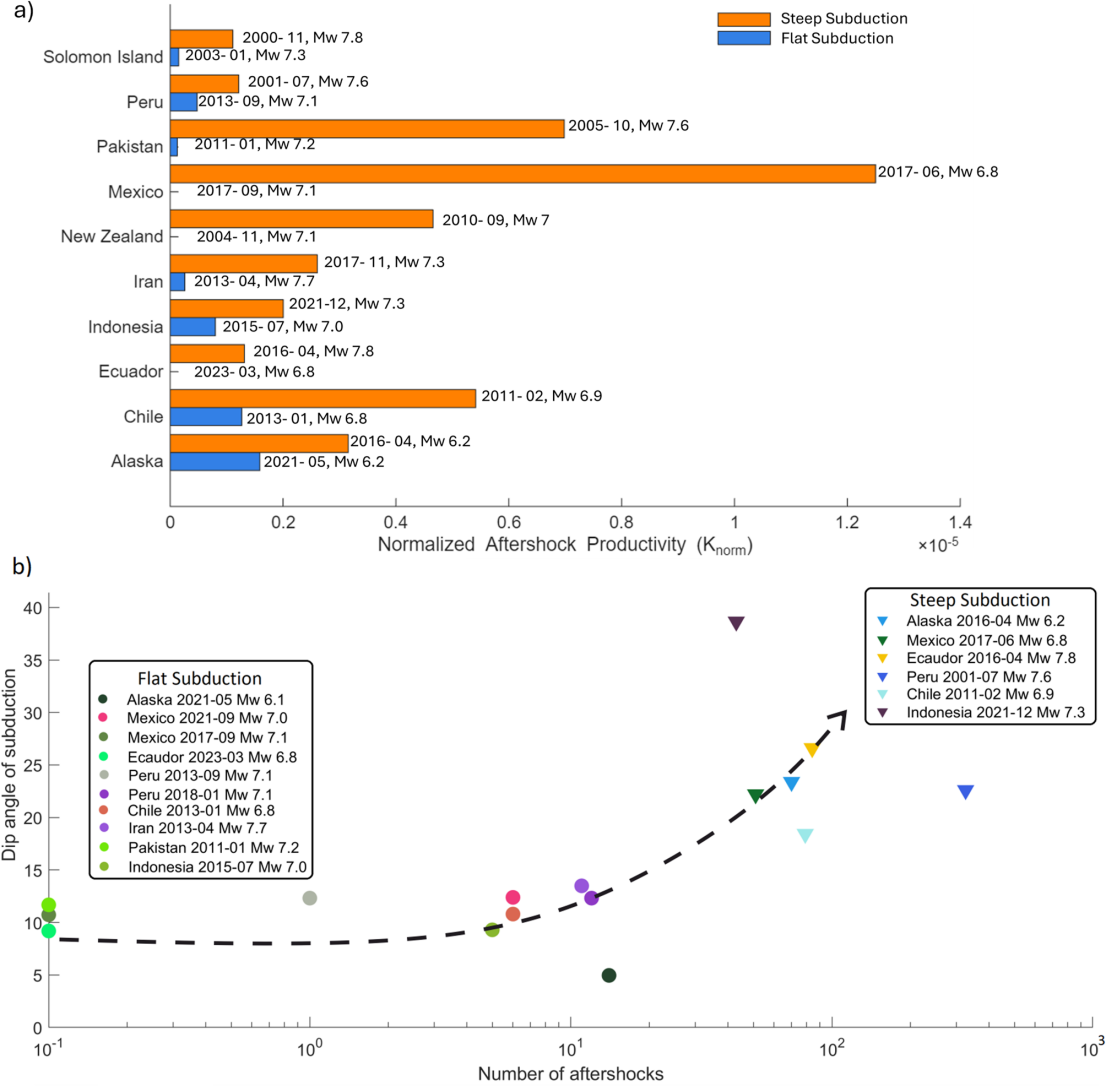
(**a**) Normalized aftershock productivity ($K_{\text {norm}}$) for earthquakes of similar magnitude. Orange bars indicate typical steep subduction earthquakes; blue bars represent events in and around flat-slab regions. (**b**) A clear logarithmic correlation is observed between the number of aftershocks (in the first three weeks) and the angle of the subducting plate, which are based on previous slab-geometry studies^66–68^.

Figure 4a compares aftershock productivity for earthquakes of similar magnitude occurring in geographically comparable settings. To enable meaningful comparison across events of different magnitude, we computed a normalized productivity metric following established approaches^64,65^, $K_{\text {norm}} = \frac{N_{\text {AS}}}{10^{\alpha M_w}},$ where $N_{\text {AS}}$ is the number of aftershocks with $M_w \ge 4.0$ within three weeks of the mainshock and $\alpha \approx 1$. This normalization removes the first-order magnitude dependence and highlights systematic differences between tectonic settings. Orange bars represent aftershocks associated with typical steep-slab earthquakes, whereas blue bars correspond to events occurring within or adjacent to flat-slab regions. Despite similar magnitudes, aftershock productivity varies substantially: some earthquakes generate abundant aftershocks, whereas others produce only a few or none.

Figure 4b demonstrates that aftershock productivity varies systematically with slab dip. For each earthquake, the local dip was extracted from regional slab-geometry models^66–68^. Flat-slab regions consistently exhibit low aftershock productivity (0–10 events with (>Mw 4.0), whereas steeper slabs commonly generate far larger sequences.

These patterns point to two dominant controls: slab dip, which governs the amount and continuity of hydrated minerals along the interface, and rupture geometry, which determines the degree to which an earthquake accesses these hydrated domains. Steep slabs preserve a continuous, mineralogically hydrated shear zone that forms the largest accessible reservoir of hydrous phases during shallow rupture. Interface-parallel ruptures in such settings can dehydrate substantial volumes of hydrous minerals and release significant overpressured fluids. In contrast, oblique or intraslab ruptures intersect much smaller and less continuous hydrated regions, generating correspondingly limited fluid release and fewer aftershocks. From a petrological perspective, these observations are consistent with global relationships between slab dip, thermal structure, and hydrous mineral stability^10,11^. Steep slabs maintain cooler conditions that stabilize hydrous phases, whereas flat slabs are thermally warmer at comparable depths and retain less bound water. Shear heating during rupture further promotes thermal dehydration of minerals such as serpentine, chlorite, and smectite^21^, enhancing fluid release where hydrous phases remain intact.

**Fig. 5:**
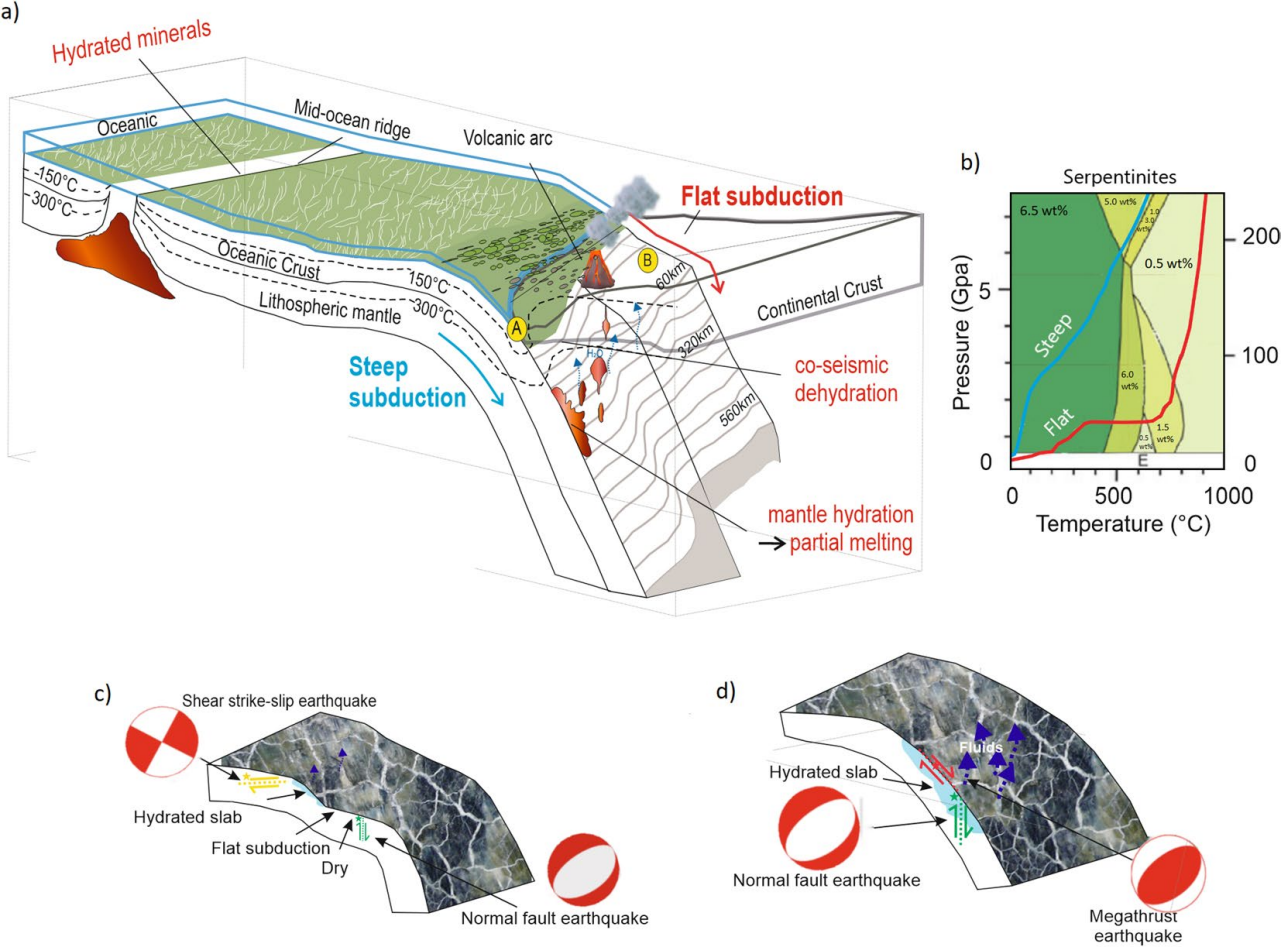
(**a**) Three-dimensional conceptual model illustrating the evolution from mid-ocean ridge (MOR) hydration to (A) steep and (B) flat subduction geometries. (**b**) Petrological perspective on the P–T paths of serpentinite in steep (blue) and flat (red) subduction zones, illustrating differences in mineral stability and water availability (modified after^77^). (**c**) Close-up view of earthquake types investigated in flat-slab regions, all of which generated very limited aftershock sequences. (**d**) Typical steep-slab subduction showing interface-parallel rupture along a hydrated mineral shear zone, which produces rich aftershock sequences through coseismic devolatilization. The schematic was drawn by the authors for this study.

Figure 5a introduces a conceptual framework that places these observations within the broader subduction-system context. Hydrothermal circulation at mid-ocean ridges generates a heterogeneous distribution of hydrated crust and upper mantle, which is subsequently transported into subduction zones. Additional hydration occurs where the accretionary prism and subducted sediments contribute hydrous minerals to the interface. Regional variations in convergence rate, plate age, and lithospheric thickness further influence thermal and mechanical structure^69,70^, producing heterogeneity in rheological and hydrological properties^71,72^. Quantitative estimates of mineralogical water content (Fig. 5b) for Serpentinite show that steep slabs contain significantly more bound water (3.3–6.5 wt.$\%$) than flat slabs (0.5–3.3 wt.$\%$), reinforcing the interpretation that slab dip strongly modulates fluid availability during rupture. Corresponding water contents for sediments and metabasalt across relevant pressure–temperature conditions are provided in the Supplementary Material (Fig. S3).

These observations allow us to distinguish two end-member rupture types: interface-parallel ruptures and intraslab ruptures. Figures  5c, d summarize the faulting styles represented in our dataset. Figure 5c includes (i) strike-slip earthquakes (yellow) that accommodate transform motion at the lateral transition between steep and flat slabs (e.g., Ecuador); (ii) normal-faulting events (green) that occur obliquely to the interface (e.g., Mexico, Peru, Alaska); and (iii) reverse-fault intraslab earthquakes (e.g., Chile, Iran–Pakistan, New Zealand, Indonesia, Solomon Islands, Peru). All of these events generated few, if any, aftershocks—consistent with their limited access to continuous hydrated domains.In contrast, Fig. 5d shows typical (iv) megathrust earthquakes rupturing along the subduction interface (red) and (v) normal-fault events within hydrated portions of the subducting plate (green), both of which commonly produce abundant aftershock sequences. The intraslab earthquake events presented in this study can be conceptualized as shown in Fig. 5d, where rupture propagates within hydrated portions of the downgoing plate, allowing access to hydrous minerals and associated fluid release despite not being interface-parallel.

These contrasting behaviors point to a common underlying mechanism. In our conceptual understanding, a rheologically weak, hydrated mineral network is embedded within stronger, less hydrated lithological domains. These stronger regions act as asperities capable of sustaining substantial shear stresses along the interface. High slip velocities generate frictional heating that thermally decomposes hydrous minerals, while further enhancement of co-seismic fluid pressurization may arise from visco-plastic compaction^73,74^ and/or thermal pressurization^75^ of pre-existing fluid-filled pores. Such pressurization processes are likely active along a heterogeneously hydrated subduction interface, where slow interseismic slab dehydration continuously supplies fluid-filled pore space^76^.

## Conclusions

Our results demonstrate that aftershock productivity in subduction zones is fundamentally controlled by access to hydrous minerals during rupture. We examined 11 earthquakes, spanning thrust, normal, and strike-slip mechanisms, that generated few, if any, aftershocks with Mw > 4.0. These included three large earthquakes, eight major earthquakes, and one great earthquake reported previously. We also examined ten earthquakes nearby of similar magnitude that generated rich and long-lasting aftershock sequences. These events included three large earthquakes and six major earthquakes. For each case, we compared the aftershock behavior and found a recurring pattern. Namely, we found significant variations in aftershock generation between steep and flat subduction, with earthquakes in steep subduction generating orders of magnitude more aftershocks than earthquakes associated with flat subduction. This contrast reflects systematic differences in the availability and access to hydrous minerals during earthquake rupture.

Slab dip governs the amount and spatial coherence of hydrous minerals along the plate interface, while rupture geometry determines whether an earthquake remains within this hydrated shear zone or cuts obliquely through the downgoing slab. In steep subduction, interface-parallel rupture promotes coseismic dehydration of hydrous minerals, generating substantial high-pressure fluids that sustain prolonged aftershock sequences. In flat subduction, by contrast, the causative faults rupture at angles oblique to the hydrated interface, intersecting only limited hydrated domains and thereby suppressing fluid generation and aftershock activity. This interpretation is consistent with recent evidence that the subduction interface comprises a multifault network rather than a single planar surface^78^.

One notable exception in our dataset—an Mw 7.3 earthquake in a carbonate platform in Iran—generated an unusually intense aftershock sequence despite the absence of oceanic lithosphere. We propose that rapid thermal decomposition of carbonates and the release of supercritical CO$_2$ played a similar role to devolatilization of hydrous minerals, consistent with observations from the Apennines^17^. Furthermore, recent evidence from intermediate-depth earthquakes^79^ indicates that fluid-driven aftershocks arise across a wide range of tectonic environments, reinforcing the broader significance of coseismic devolatilization.

From our results, we propose a testable hypothesis that thermal decomposition and/or a fluid source at depth is the primary driver of rich aftershock sequences. Future work will test this hypothesis by modeling slab thermal structures, mapping mineral stability fields, and quantifying volumetric fluid release for both steep and flat subduction geometries.

## Supplementary Information

Supplementary Information.

## Data Availability

The seismic data was obtained from the International Seismological Centre (ISC) (https://www.isc.ac.uk/iscbulletin/search/catalogue/), where one can use the search function to filter data based on parameters such as time period, event magnitude, depth, and geographic location. One can download the data in various formats such as CSV or ASCII for further analysis. Slab depth information are taken from the US Geological Survey data release, https://doi.org/10.5066/F7PV6JNV.
