# Supplementary material for: Rupture access to hydrous minerals controls aftershocks in subduction zones
Source: Sci Rep. 2026 Feb 10;16:8109. doi: 10.1038/s41598-026-38159-6 (PMC12960787; doi:10.1038/s41598-026-38159-6)
Supplement: Supplementary file 1 — Supplementary Information. [file 41598_2026_38159_MOESM1_ESM.pdf]

Rupture access to hydrous minerals controls aftershocks in subduction zones

Thanushika Gunatilake[1,2*], Taras Gerya[2], James A. D. Connolly[2], Stephen A. Miller[1]

[1*]University of Neuchâtel, The Centre for Hydrogeology and Geothermics (CHYN), Rue Emille Argand 8, Neuchâtel, 2000, Neuchâtel, Switzerland.
[2]ETH Zürich, Department of Earth Sciences, Sonnengesstrasse 5, Zürich, 8006, Zürich, Switzerland.

*Corresponding author(s). E-mail(s): thanushika.gunatilake@sed.ethz.ch;
Contributing authors: taras.gerya@erdw.ethz.ch; james.connolly@erdw.ethz.ch; stephen.miller@unine.ch;

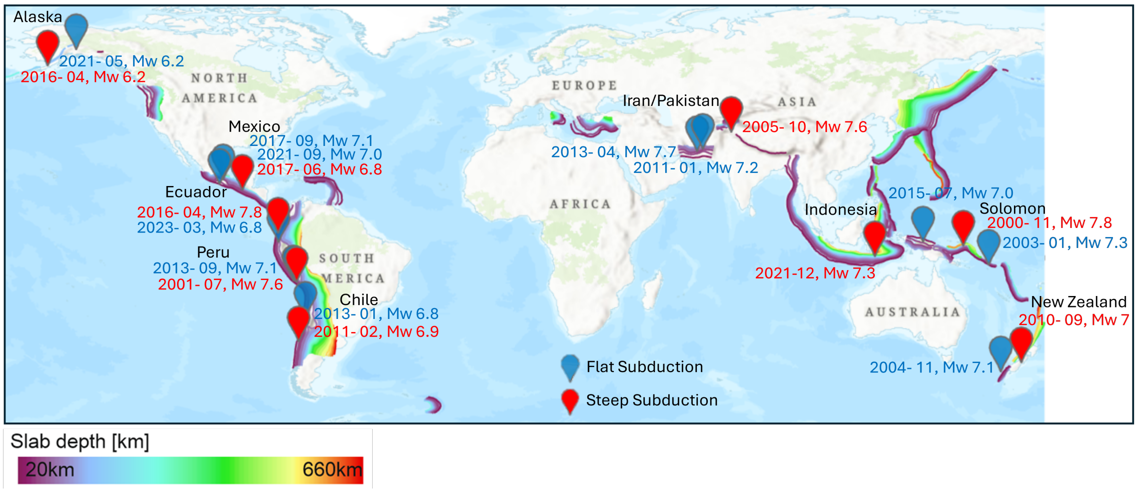

Fig. S1 Global overview of all earthquakes analyzed in this study. Colored contours indicate slab depth (20–660 km) based on the Slab2.0 model. Red markers denote earthquakes occurring in regions of steep subduction, while blue markers indicate events associated with flat-slab or near–flat-slab segments. For each location, event dates and moment magnitudes (Mw) are labeled. The figure illustrates the global distribution of paired aftershock-productive and aftershock-poor earthquakes across different subduction geometries that form the basis of this study. Maps were generated by the authors using ArcGIS Pro (v3.1; https://www.esri.com/arcgis-pro).

An Mw 6.2 steep subduction earthquake in Alaska (04/2016) generated 70 aftershocks Mw 4 in within first three weeks (Sup. Figure 2a, Panel 1). Towards the east in the flat subduction region, an earthquake of similar magnitude (Mw 6.1) stimulated a single aftershock (Sup. Figure 2a, Panel 2). The focal sphere at depth shows that this earthquake, like Peru, Mexico, and Pakistan, ruptured obliquely to the hydrated subducting interface.

In the Soloman Islands, a major Mw7.8 (11/2000) subduction zone earthquake produced more than 690 aftershocks Mw4 in the first three weeks (Sup. Figure 2b, Panel 1). Roughly 2 years later, a major (Mw 7.3) earthquake towards the southeast (Sup. Figure 2b, Panel 2) generated three aftershocks $M_w \leq 4$ in the first weeks. Slab depth indicates slab break-off at around 160km, eliminating the slab-pull driving mechanism, and leaving only regional compressional forces to drive this thrust event. The focal mechanism (S Figure 2c) shows that this earthquake ruptured almost perpendicular to the direction of subduction, consistent with the location of the aftershocks and local plate motion vector. Therefore, this earthquake ruptured the oceanic lithosphere, and not the plate interface, converging again to limited sampling of hydrous minerals for thermal decomposition. Furthermore, the volcanic arcs at this location strongly indicates dehydration never occurs (see Figure 1d in main text).

A situation identical to the Soloman Islands is found in New Zealand where a Mw7 thrust earthquake (09/2010) spawned 126 aftershocks Mw 4 within the first 3 weeks (Sup. Figure 2c, Panel 1), while a larger magnitude earthquake Mw7.1 further South generated no aftershocks (Sup. Figure 2c, Panel 1). Subduction in the south of New Zealand involves the Australian continental plate being subducted beneath the Pacific plate by the highly compressive tectonics. In addition, the focal mechanism oblique to the direction of subduction, and in line with the local plate motion vector, further indicating limited sampling of hydrous minerals during earthquake rupture. The hypocenter of the aftershock rich New Zealand earthquake likely ruptured hydrous bearing minerals in greywacke, providing a fluid source through thermal decomposition.

Supplemental Figure 3 shows the water content of serpentine as a function of PT superposed to the PT path of flat and steep subduction. These observations suggest a major source of fluid at around 500°C releasing, in order, 3 wt % (sediments) 2.5 wt % (metabasalt), and 6.5 wt % serpentine for steep subduction. Meanwhile, the PT path experienced during flat subduction follows through less hydrous mineral assemblages with 1.5 wt % (sediments), 0.5 % (metabasalt), and 6 wt % serpentine for flat subduction.

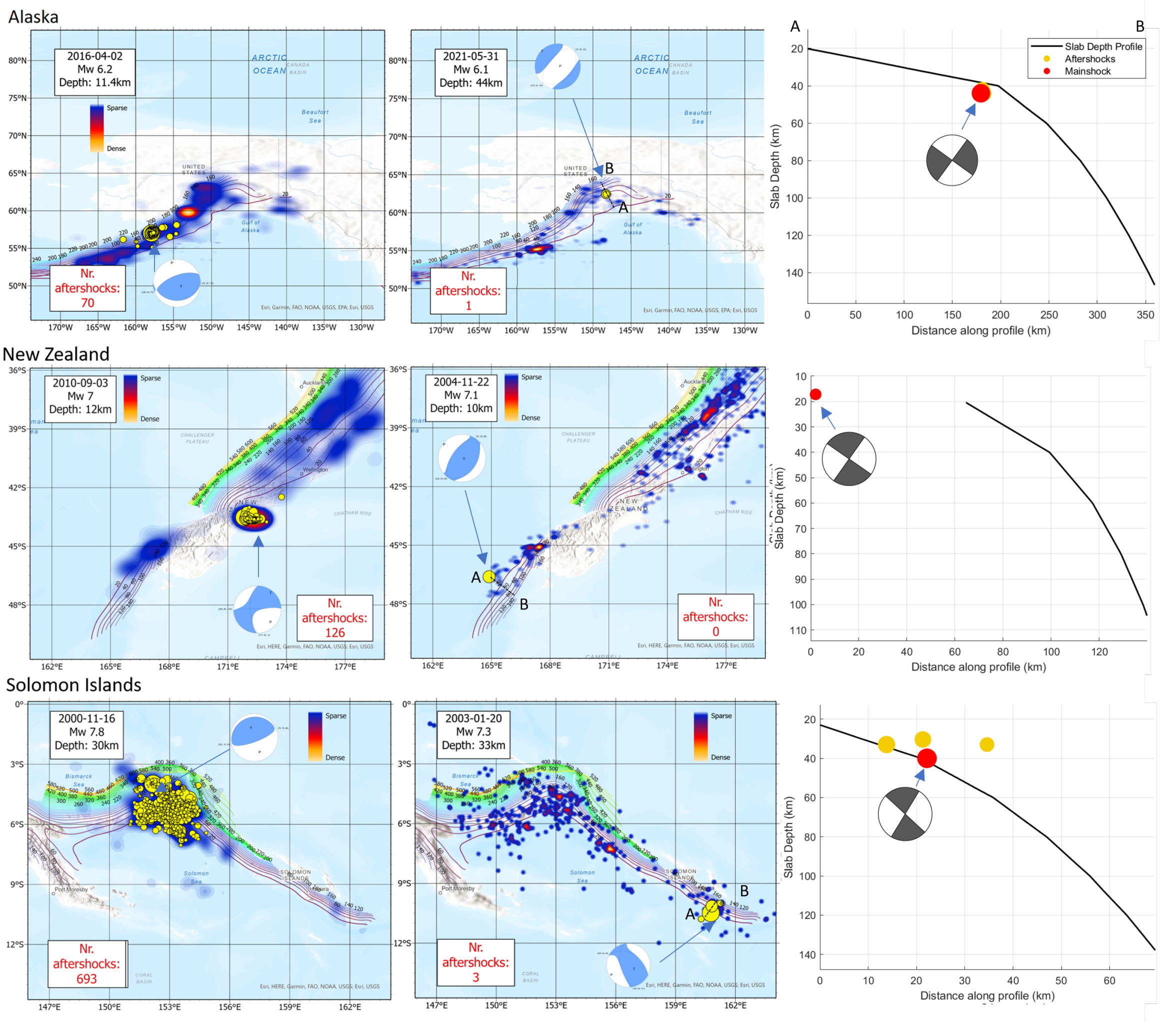

Fig. S2 Earthquakes studied: Alaska, New Zealand, Solomon Islands with slab depth superposed. In Panel 1, we observe three significant events that have resulted in extensive aftershock sequences (shown as heat maps of aftershock density). Panel 2, on the other hand, shows major seismic events that generated few aftershocks. Panel 3 shows 2D profile of the slab interface and the focal sphere at depth, showing that the earthquakes rupture oblique to this interface. New Zealand is continental subduction beneath the Pacific plate, so is a unique situation. See text for detailed description. Maps were generated by the authors using ArcGIS Pro (v3.1; https://www.esri.com/arcgis-pro) based on publicly available seismic data from the International Seismological Centre (ISC; https://www.isc.ac.uk/) and slab depth models from the U.S. Geological Survey (Hayes et al., 2018 [1]; https://doi.org/10.5066/F7PV6JNV)

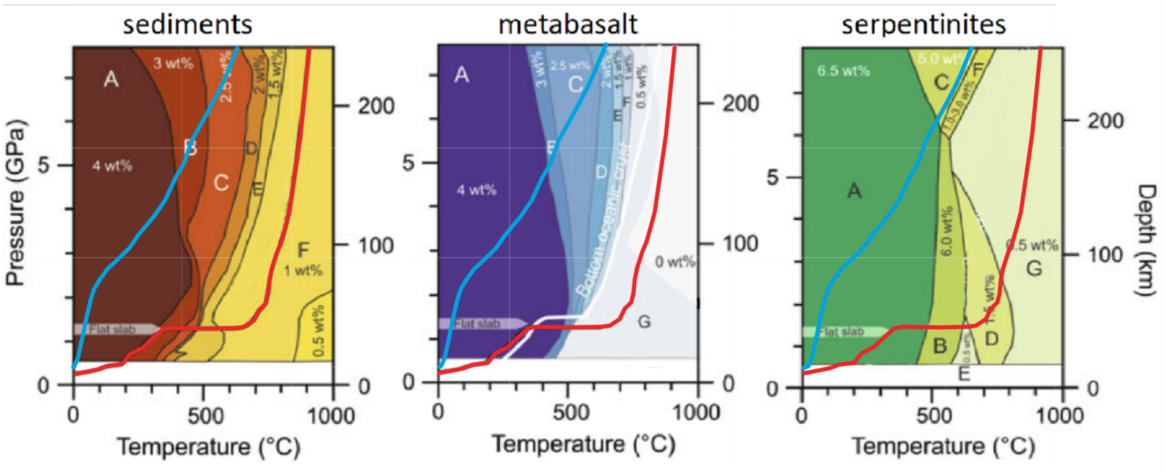

Fig. S3 a) Water content at different pressures and temperatures for sediments (a), sediments (b), metabasalt (c) serpentinites. Modifed after [2]. The PT path for flat subduction (red line) shows very little potential for fluid production, while the PT path for steep subduction (blue line) indicates substantial fluid production and which feeds volcanic arcs.

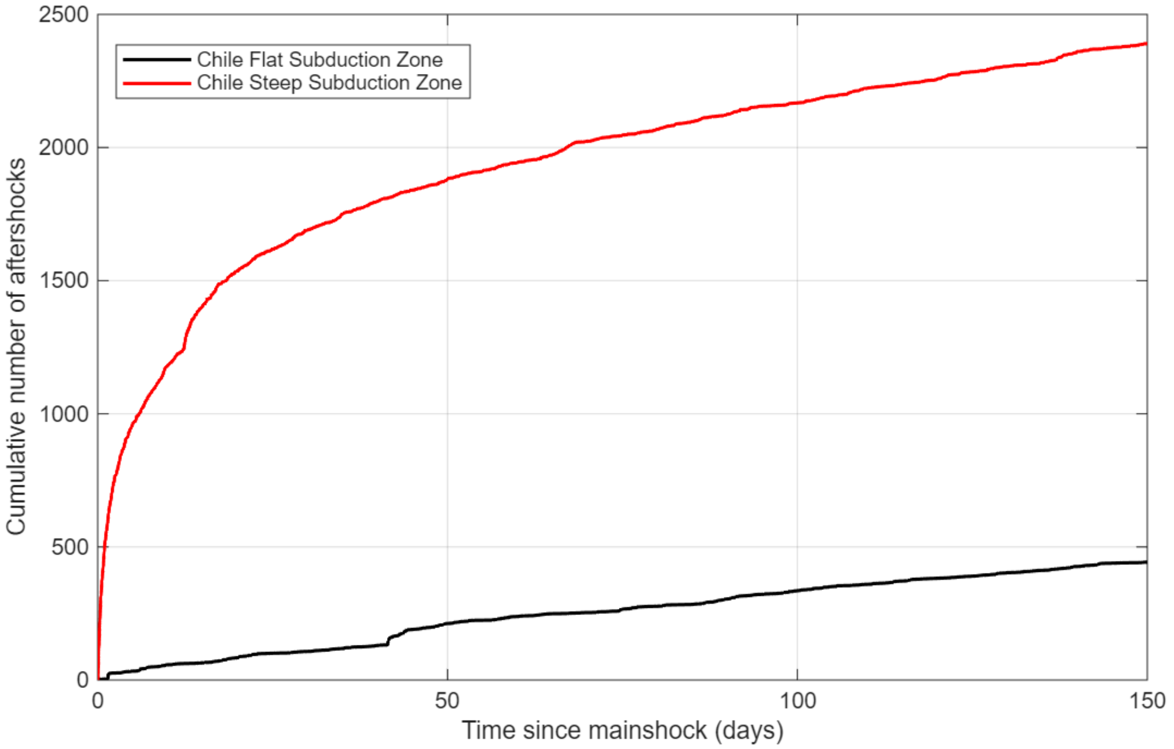

Fig. S4 Cumulative aftershock sequences for two Chilean earthquakes illustrating the contrast between flat-slab and steep-slab subduction settings. The steep-slab event (red) produced more than 2,300 aftershocks within 150 days, whereas the flat-slab event (black) generated fewer than 500 aftershocks over the same time period.

References

[1] G. Hayes. *Slab2-A Comprehensive Subduction Zone Geometry Model: US Geological Survey data release, doi: 10.5066*. 2018.

[2] V. C. Manea and M. Manea. "Flat-slab thermal structure and evolution beneath central Mexico". In: *Pure and Applied Geophysics* 168 (2011), pp. 1475–1487.
